# Examining longitudinal associations between interpersonal outcomes and general psychopathology factors across preadolescence using random intercept cross‐lagged panel model

**DOI:** 10.1111/jcpp.14105

**Published:** 2024-12-28

**Authors:** Tom Chin‐Han Wu, Alex Lloyd, Essi Viding, Pasco Fearon

**Affiliations:** ^1^ Department of Clinical, Educational and Health Psychology University College London London UK; ^2^ Department of Psychology, Centre for Family Research University of Cambridge Cambridge UK

**Keywords:** Friendship quality, popularity, p‐factor, RI‐CLPM

## Abstract

**Background:**

Interpersonal outcomes and mental health problems are closely associated. However, their reciprocal influence has not been directly examined while considering the temporal stability of these constructs, as well as shared and unique variance associated with internalising, externalising and attention problems. Using random intercept cross‐lagged panel models (RI‐CLPM), we tested the hypotheses that negative bidirectional associations at the between‐person and negative cross‐lagged effects at the within‐person level would emerge between interpersonal outcomes (friendship quality and perceived popularity) and mental health problems (i.e. general psychopathology factor) during preadolescence.

**Methods:**

Participants (*n* = 918) were from the NICHD Study of Early Child Care and Youth Development. Psychopathology, consisting of a general psychopathology factor (p‐factor) and three specific factors (internalising, externalising and attention problems), was derived from mother‐reported Child Behaviour Checklist symptoms. Friendship quality was assessed using the self‐reported Friendship Quality Questionnaire. Popularity was assessed using teacher‐reported popularity ranking. Four RI‐CLPM were estimated to examine the associations between interpersonal outcomes and psychopathology at between‐ and within‐person levels across four timepoints (mean ages 8–11).

**Results:**

At the between‐person level, popularity scores, but not friendship quality, were negatively associated with p‐factor scores (β = −.33). At the within‐person level, we found (i) p‐factor scores at age 9 negatively predicted friendship quality and popularity at age 10, but not at other ages (β = −.16 to −.19); (ii) specific externalising factor scores at age 10 negatively predicted friendship quality at age 11 (β = −.10) and specific internalising factor scores at ages 8 and 9 positively predicted friendship quality at ages 9 and 10 (β = .09–.12) and (iii) popularity at age 10 negatively predicted specific internalising factor scores at age 11 (β = −.12).

**Conclusions:**

Psychopathology was found to influence interpersonal outcomes during preadolescence, while the reverse effects were less readily observed, once between‐person level effects were accounted for.

## Introduction

Interpersonal outcomes and mental health difficulties are thought to reciprocally influence each other, particularly as children grow up and gain independence (Bernasco, Nelemans, van der Graaff, & Branje, 2021; Waldrip, Malcolm, & Jensen‐Campbell, 2008). From age 8 onwards, children tend to spend increasingly more time with peers (Lam, McHale, & Crouter, 2014), and their importance more generally increases over development, while that of parents decreases (Andrews, Ahmed, & Blakemore, 2021). Accordingly, associations have been identified during preadolescence between important aspects of interpersonal outcomes (e.g. friendship quality and perceived popularity) and mental health problems (Sandstrom & Cillessen, 2006; Waldrip et al., 2008). However, their reciprocal influences have not been directly examined across this period (e.g. via cross‐lagged panel analysis), despite data and theory both pointing strongly towards the likelihood of such reciprocal influences. Moreover, past research has rarely examined general (and more specific domains of) psychopathology in relation to peer relationships, although extant findings have demonstrated that associations between peer relationships and psychopathology are not limited to certain disorders (e.g. depression or ADHD; Litwack, Aikins, & Cillessen, 2012; Normand, Miller, & Mikami, 2023) or subdomains of psychopathology (e.g. internalising and externalising problems; Waldrip et al., 2008). Therefore, the aim of this study was to examine the reciprocal associations between important interpersonal outcomes (i.e. friendship quality and perceived popularity) and mental health problems in general during preadolescence.

### Friendship quality and mental health problems

Friendship quality, as opposed to the *quantity* of friendships or time spent with friends, is an important interpersonal outcome (Berndt, 1982; Hartup & Stevens, 1997) and has been shown to be negatively associated with internalising, externalising and attention problems during preadolescence (e.g. Bernasco et al., 2021; Schwartz‐Mette, Shankman, Dueweke, Borowski, & Rose, 2020; Waldrip et al., 2008). These associations could emerge for several reasons. For example, friends provide emotional support, companionship and advice in difficult situations, alleviating mental health symptoms (van Harmelen et al., 2016). Here, in a sample of 238 adolescents (age 12), Waldrip et al. (2008) found that higher self‐reported friendship quality was associated with less teacher‐rated internalising (β = −.22) and externalising problems (β = −.17) later in the school year, even after controlling for friendship *quantity*. Similarly, negative friendship quality at age 12 has also been found to be associated with higher levels of depressive symptoms at age 15 (*r* = .08; Kamper & Ostrov, 2013). A recent meta‐analysis also found concurrent and prospective associations between depression and positive (*r* = −.26 to −.41) and negative (*r* = .21) aspects of friendship quality (Schwartz‐Mette et al., 2020). On the other hand, individuals suffering from mental health problems may have poorer social skills (Bornstein, Hahn, & Haynes, 2010) or behave in ways that alienate their peers (Salvas et al., 2011). In a sample of children (age 6–11), Normand et al. (2023) found that higher levels of externalising problems and ADHD symptoms were associated with negative friendship quality (β = −.17). A recent meta‐analysis also found that aggressive behaviours were associated with poorer friendship quality (*r* = .19; mean age 11.7 years; Dryburgh, Ponath, Bukowski, & Dirks, 2022). Finally, preadolescents experiencing mental health problems may also have a more negative outlook (Kingery, Erdley, Marshall, Whitaker, & Reuter, 2010) and anticipate more problems and rejection in peer relationships (e.g. Hannesdottir & Ollendick, 2007a; London, Downey, Bonica, & Paltin, 2007) and thus become more likely to withdraw from peers (Biggs, Vernberg, & Wu, 2012). Accordingly, self‐ and parent‐reported internalising problems in preadolescents (mean age 11.6 years) have been found to predict lower friendship quality 6 months later (β = −.16; Bernasco et al., 2021).

While most studies report negative associations between friendship quality and mental health problems, some studies reported no such associations (e.g. Dirghangi et al., 2015; Gallagher, Prinstein, Simon, & Spirito, 2014).

### Perceived popularity and mental health problems

Another important interpersonal outcome for children and adolescents is to be seen as popular among peers (i.e. *perceived* popularity; de Bruyn & van den Boom, 2005). Indeed, being popular and having a higher social position are of primary concern for children and adolescents (Adler & Adler, 1996; Parker & Gottman, 1989). The literature also suggests that popularity is associated with internalising, externalising and attention problems during preadolescence (e.g. Arango‐Tobón, Guevara Solórzano, Orejarena Serrano, & Olivera‐La Rosa, 2023; Litwack et al., 2012; Rose, Swenson, & Waller, 2004). For instance, popular children may feel more confident and socially successful, thus experiencing fewer internalising symptoms (Prinstein & La Greca, 2002; Sandstrom & Cillessen, 2006). Conversely, those with internalising problems could be more withdrawn and may have difficulties approaching peers, resulting in fewer friendships (Eggum‐Wilkens, Valiente, Swanson, & Lemery‐Chalfant, 2014) and thus are less likely to be perceived as popular. Indeed, in a sample of 245 eighth graders in the US, Litwack et al. (2012) found that higher perceived popularity was associated concurrently with lower depressive affect (β = −.22). Moreover, in a sample of 1974 students (ages 12–13), anxious students were more likely to be nominated as being rejected and unpopular (Favre, Aksoy, Janousch, & Garrote, 2022). Relatedly, the literature suggests that perceived popularity is associated with externalising behaviours positively during adolescence but negatively earlier in development. Adolescents engaging in externalising behaviours could be perceived as dealing more successfully with the ‘maturity gap’ (Franken, Harakeh, Veenstra, Vollebergh, & Dijkstra, 2017; Moffitt, 1993), thus gaining higher social status. This is not the case for younger children, however, because they may lack the interpersonal skills to display externalising problems (e.g. aggression) strategically to appear socially dominant (Rose et al., 2004). Indeed, Rose et al. (2004) found that externalising behaviours such as overt aggression were associated positively with perceived popularity among year 9 students (*r* = .18) but negatively among year 3 students (*r* = −.27). Finally, attention problems (e.g. ADHD) could also be negatively associated with perceived popularity, although this has not been directly examined. Here, prosocial children have been found to be more popular (γ = .64; Meisinger, Blake, Lease, Palardy, & Olejnik, 2007) but children with ADHD tend to have deficits in prosocial behaviours (Arango‐Tobón et al., 2023).

### Reciprocal associations between peer relationships and psychopathology

The literature thus demonstrates that friendship quality and perceived popularity are closely associated with mental health problems; however, two important limitations exist in prior research.

First, most studies did not directly test reciprocal associations or take into account stable between‐person components of these constructs. To address these issues, we estimated random intercept cross‐lagged panel models (RI‐CLPM; Hamaker, Kuiper, & Grasman, 2015), an extension of the traditional cross‐lagged panel models (CLPM). Apart from examining reciprocal (i.e. cross‐lagged) associations like CLPM, RI‐CLPM further captures and separates stable between‐person effects using random intercepts. In RI‐CLPM, between‐person associations answer questions such as, do participants who report lower friendship quality scores also have, on average, higher p‐factor scores across timepoints, compared to participants who report higher friendship quality. Controlling for these trait‐like effects is crucial because these effects, sometimes caused by unmeasured stable confounders such as genetic vulnerabilities or environmental influences, could intercorrelate and give rise to spurious cross‐lagged associations (Allegrini et al., 2022; Masselink et al., 2018). Conversely, within‐person components reflect fluctuations across timepoints around one's own expected, average score. For example (Masselink et al., 2018), suppose a participant scores on average three points higher than the population mean at all measurement points, and the population average at Time 1 is 2, then this participant's expected score would be 2 + 3 = 5 at this timepoint. If the participant's true score is 6 at Time 1, then the deviation from the expected score would be positive 1. The within‐person latent component captures this deviation at each timepoint, allowing within‐person associations between constructs to be estimated. In RI‐CLPM, significant cross‐lagged effects thus indicate that deviation from the expected score of one construct (e.g. popularity) at one timepoint predicts deviation from another (e.g. p‐factor) at the subsequent timepoint. Such within‐person variations are thought to be less impacted by confounders and provide more powerful evidence of potential causal influence over time (Masselink et al., 2018). Similarly, significant concurrent associations indicate that deviations from the expected score of one construct are concurrently associated with those of another. This distinction between effects at different levels is especially important when exploring processes that could be within‐person in nature. Here, one's own friendship quality and popularity are hypothesised to portend risks to one's own mental health problems, where risks could increase during or following periods of turbulence in friendship quality or lower popularity and vice versa. To note, findings at the within‐ and between‐person levels can also be different (e.g. Dietvorst, Hiemstra, Hillegers, & Keijsers, 2018), which could help explain some discrepancies observed in the literature. In other words, RI‐CLPM thus allows for a more nuanced examination of the relationship between interpersonal outcomes and mental health, enabling us to investigate both between‐ and within‐person hypotheses.

Second, most studies examining peer relationships and psychopathology have focused on discrete diagnoses (e.g. depression; Litwack et al., 2012) or subdimensions of psychiatric disorders (e.g. internalising problems; Bernasco et al., 2021). However, taken together, past research clearly indicates that it may be a general risk for psychopathology shared across different disorders and subdimensions of psychopathology, as opposed to risk specific to particular disorders or subdomains, that may account for associations between peer relationships and mental health—this has not been formally tested. Moreover, mental health problems also tend to co‐occur (Kotov et al., 2017), among closely related disorders (e.g. depression and anxiety), as well as those across diagnostic domains (e.g. depression and conduct disorder), suggesting that there may be general vulnerability to multiple forms of psychopathology. To account for this general vulnerability, therefore, we included a bifactor model of psychopathology in the present study, which consisted of a general psychopathology factor that confers overall liability to mental health problems (p‐factor; Caspi et al., 2014; Lahey et al., 2012) and three specific factors of internalising, externalising and attention problems and examined the associations between peer relationships and these different factors.

### Current study

Using longitudinal data from students aged 8–11, this pre‐registered study (https://osf.io/e8xdu/?view_only=bb21c684c4df4f8a96f43fffea75f4c2) examined the reciprocal associations between interpersonal outcomes (friendship quality and perceived popularity) and mental health problems. This age range was chosen because assessments done during this period are potentially more accurate and ecologically valid than those during later timepoints. Children at these ages spend the majority of their time in self‐contained classrooms, providing valid assessments of their friendship experiences. Also captured in our data is the transition from elementary to middle school for American preadolescents, when interpersonal experiences could become increasingly important (Berndt, 1979).

We tested three main hypotheses: First, friendship quality and popularity would be negatively associated with the p‐factor and the specific internalising, externalising and attention factors at the between‐person level (i.e. negative bidirectional associations demonstrating between‐person processes). Second, at the within‐person level, higher‐than‐expected levels of psychopathology (p‐factor and specific factors) would predict *lower‐*than‐expected levels of friendship quality and perceived popularity (i.e. negative cross‐lagged effects from psychopathology to interpersonal outcomes). Finally, also at the within‐person level, higher‐than‐expected friendship quality and popularity would predict *lower‐*than‐expected levels of psychopathology (i.e. negative cross‐lagged effects from interpersonal outcomes to mental health problems).

## Methods

### Sample

The Study of Early Child Care and Youth Development (SECCYD) was a longitudinal study initiated by NICHD to investigate the interrelations among childcare experiences, childcare characteristics and developmental outcomes of children (NICHD Early Child Care Research Network, 2005). Data collection began in 1991 at 10 different sites across the US, with the initial cohort consisting of 1,364 parent–child pairs. The present study included 918 participants (50% female) who had psychopathology symptoms and peer relationship data were collected yearly between years 3 and 6 (age 8–11 years). Ethical approval for the SECCYD was granted by the data‐collecting universities, and informed consent was obtained at each assessment. Further details on the study are available on the NICHD website (https://www.nichd.nih.gov/research/supported/seccyd/overview#instruments).

### Measures

*Psychopathology* was assessed using mother‐reported Child Behaviour Checklist (CBCL; Achenbach, Dumenci, & Rescorla, 2001). Mothers reported on their child's internalising (*n* = 24; e.g. *Cries a lot*), externalising (*n* = 19; e.g. *Gets in many fights*) and attention (*n* = 8; e.g. *Can't sit still, restless or hyperactive*) problems in the past 6 months on a 3‐point scale ranging from 0 (‘not true’) to 2 (‘very true/often’). Consistent with prior research, items were recoded to indicate the absence (‘0’) or presence (‘1’) of symptoms, given low endorsement rates of responses indicative of severe symptomology (Achenbach et al., 2001; McElroy, Belsky, Carragher, Fearon, & Patalay, 2018). Items were included in the analysis if at least 5% of individuals indicated having the symptom at all four timepoints. All subscales demonstrated acceptable reliability (α = .74–.88). A latent bifactor model of psychopathology, consisting of a general psychopathology factor (p‐factor), and three orthogonal specific factors (internalising, externalising and attention factors) was estimated using these items, and the factor scores were saved and included in subsequent analysis. The use of saved factor score is consistent with prior research (McElroy et al., 2018) and has the advantage of modelling measurement at the latent level (Bollen, 1989; DiStefano, Zhu, & Mîndrilă, 2009) that is particularly important for the p‐factor.

*Friendship quality* was assessed using the Friendship Quality Questionnaire (Parker & Asher, 1993). Participants reported on their perceived friendship quality with a particular best friend, on a 5‐point scale ranging from 1 (‘not at all true’) to 5 (‘really true’). The 29‐item questionnaire assesses the dimensions of Validation and Caring (e.g. *Tells me I am good at things*), Help and Guidance (e.g. *Loan each other things all the time*), Companionship and Recreation (e.g. *Always sit together at lunch*), Intimate Exchange (e.g. *Tell each other private things a lot*), Conflict (e.g. *Argue a lot*) and Conflict Resolution (e.g. *Always makeup easily when we have a fight*). All items are re‐coded such that higher scores indicate higher friendship quality, and the sum score at each timepoint was used in the analysis. The measure demonstrated good internal reliability across timepoints (α = .86–.90).

*Perceived popularity* was assessed using teacher‐rated popularity ranking of the study child among same‐sex classmates. At each timepoint, teachers were asked to make a list of every same‐sex child in the classroom as the study child. Then, teachers ranked the children based on their popularity with classmates. Popularity was then calculated by subtracting the study child's ranking from the total number of same‐sex children in the class plus 1, then divide by the total number of same‐sex children in the class. Higher scores indicate higher popularity, with a maximum score of 1. In other words, popularity reflects each study child's unique ranking in class, and the within‐person effects represent deviations from one's expected popularity score.

*Gender* of the study child was included as a time‐invariant covariate in all models by regressing the between‐person components on gender.

### Statistical analysis

The analysis was conducted in two steps. First, confirmatory factor analyses were conducted to examine the factor structure of psychopathology at the four timepoints. Consistent with McElroy et al. (2018), bifactor models were specified with the p‐factor (i.e. the general psychopathology factor) and three orthogonal specific factors of internalising, externalising and attention problems and estimated using the weighted least squares mean and variance adjusted (WLSMV) estimator. Correlations among all factors (general and specific) were fixed to zero. To further assess the fit of the bifactor models, three bifactor‐specific indices were considered: (i) omega (*ω*), a model‐based reliability coefficient, (ii) explained common variance (ECV), the proportion of common variance explained by a given factor and (iii) construct replicability (H), how well the latent construct is represented by its items (Dueber, 2017; Hancock & Mueller, 2001).

Second, four RI‐CLPM were estimated to examine the bidirectional and reciprocal associations between the interpersonal outcomes and mental health problems at between‐ and within‐person levels. In Models 1 and 2, RI‐CLPM were estimated with either friendship quality (Model 1) or perceived popularity (Model 2) and p‐factor scores only. Between‐person variance of the interpersonal outcomes and p‐factor scores was captured by including two random intercept factors in each model. In Model 1, for example, the sum score of friendship quality and p‐factor scores from Time 1 to Time 4 were used as indicators for their respective random intercept factor, where all factor loadings were to 1. The within‐person component was then estimated by regressing the observed friendship quality and p‐factor scores on their own latent factors, with the factor loading also constrained to 1. This resulted in 8 such latent factors, one for each variable (friendship quality and p‐factor) at each time point (Time 1 to Time 4). The specific internalising, externalising and attention factor scores were additionally analysed in Models 3 (with friendship quality) and 4 (with perceived popularity). To note, the present analysis may be underpowered as sample sizes above 1,000 could be required to detect moderate effect sizes in RI‐CLP models (Masselink et al., 2018).

The four RI‐CLPM were estimated using the robust maximum likelihood estimator, and full‐information maximum‐likelihood (FIML) method was used to handle missing data. The Comparative Fit Index (CFI), Tucker‐Lewis Index (TLI) and root mean square error of approximation (RMSEA) were used to examine the goodness‐of‐fit for all models. Specifically, CFI and TLI above 0.90 (Bentler & Bonett, 1980) and RMSEA below 0.08 (Browne & Cudeck, 1993) indicate acceptable model fit. All analyses were conducted using Mplus version 8.9 (Muthén & Muthén, 2017).

## Results

### Descriptive statistics, model fit and model‐based reliability

Descriptive statistics and bivariate correlations of all variables are shown in Table 1. Model fit statistics for the bifactor models and the four RI‐CLPM are presented in Table 2; all models demonstrated adequate‐to‐excellent fit. Further goodness‐of‐fit indices for the bifactor models are presented and discussed in the (Appendix S1).

**Table 1:** Descriptive statistics and bivariate associations for study variables

	GPF8Y	GPF9Y	GPF10Y	GPF11Y	INT8Y	INT9Y	INT10Y	INT11Y	EXT8Y	EXT9Y	EXT10Y	EXT11Y	ATT8Y	ATT9Y	ATT10Y	ATT11Y	FSQ8Y	FSQ9Y	FSQ10Y	FSQ11Y	POP8Y	POP9Y	POP10Y	POP11Y
GPF8Y	1																							
GPF9Y	.80***	1																						
GPF10Y	.76***	.79***	1																					
GPF11Y	.71***	.74***	.78***	1																				
INT8Y	.14***	.16***	.13***	.12***	1																			
INT9Y	.08*	.14***	.10**	.10**	.61***	1																		
INT10Y	.08*	.17***	.15***	.14***	.55***	.58***	1																	
INT11Y	.10**	.13***	.14***	.16***	.53***	.56***	.57***	1																
EXT8Y	.23***	.19***	.18***	.18***	−.27***	−.22***	−.19***	−.20***	1															
EXT9Y	.23***	.19***	.19***	.18***	−.23***	−.20***	−.20***	−.18***	.56***	1														
EXT10Y	.20***	.17***	.17***	.21***	−.22***	−.18***	−.22***	−.22***	.50***	.53***	1													
EXT11Y	.18***	.16***	.16***	.15***	−.25***	−.24***	−.19***	−.25***	.51***	.51***	.51***	1												
ATT8Y	.07*	.09**	0.06	0.04	−.09**	−0.06	−0.03	−0.04	0.05	0.05	0.02	0.06	1											
ATT9Y	.11***	0.05	.07*	0.04	−.07*	−0.06	−.09*	−.07*	0.03	0.01	0.01	.09**	.57***	1										
ATT10Y	.09**	0.06	0.04	0.05	−0.01	−0.02	0	−0.03	0.05	0.02	0.01	0.06	.59***	.61***	1									
ATT11Y	.12***	.07*	.10**	0.06	−0.05	−0.06	−0.06	−0.06	0	0.04	0.01	0.06	.55***	.53***	.60***	1								
FSQ8Y	−.07*	−0.06	−0.03	−0.03	−0.02	0	0.02	0	−0.05	−0.05	0.01	−.07*	0.01	−0.02	.01***	−0.02	1							
FSQ9Y	−0.03	−0.01	0.01	−0.02	.08*	0.05	0.05	0.03	−0.06	−.12***	−.07*	.10**	0.01	−0.01	−0.02	−0.01	.19***	1						
FSQ10Y	0.01	0.03	0.01	−0.01	0.04	0.03	0.03	0.04	−.07*	−0.05	−0.01	−0.04	0.01	−0.01	−0.01	0.05	0.04	.14**	1					
FSQ11Y	−0.04	−0.03	−0.02	0	−0.01	0.02	0.02	0.02	−.07*	−0.03	−0.01	−0.03	−0.04	0.02	−0.06	−0.1	.08*	.12***	0.04	1				
POP8Y	−.22***	−.21***	.21***	−.21***	−0.01	0	−0.01	−0.03	−0.05	−.07*	−0.02	−0.01	−.23***	−.14***	−.16***	−.18***	−0.04	−0.02	0.01	0.04	1			
POP9Y	−.19***	−.22***	−.22***	−.24***	−0.03	−0.07	−0.01	0.01	−0.04	−0.05	−.08*	−0.05	−.16***	−.17***	−.16***	−.16***	0.01	−0.01	0.04	0.07	.47***	1		
POP10Y	−.13***	−.21***	−.19***	−.19***	−0.04	−.07*	−0.05	−.09*	−0.01	−0.04	0.01	−0.02	−.23***	−.16***	−.16***	−.19***	0	−0.01	0.01	0.04	.41***	.50***	1	
POP11Y	−.13***	−.15***	.15***	−.13***	−0.04	−0.05	−0.03	−.11**	0.05	−0.02	0.05	0.01	−.18***	−.08*	−.12**	−.11**	−0.07	−.08*	−0.05	0.03	.34***	.39***	.51***	1
Mean	0.05	0.05	0.05	0.06	0.03	0.03	−0.02	0.02	0.03	0.03	0.04	0.03	0.03	0.03	0.04	0.03	82.19	83.23	86.07	86.75	0.62	0.62	0.62	0.6
*SD*	0.86	0.87	0.87	0.86	0.74	0.72	0.73	0.73	0.64	0.65	0.63	0.63	0.67	0.68	0.64	0.63	13.13	13.55	12.09	12.93	0.24	0.24	0.24	0.23

ATT8Y, attention problems at age 8; EXT8Y, externalising problems at age 8; FSQ8Y, friendship quality at age 8; GPF8Y, general psychopathology factor at age 8; INT8Y, internalising problems at age 8; POP8Y, perceived popularity at age 8.

*p < .05; **p < .01; ***p < .001.

**Table 2:** Fit statistics of CFA across time and RI‐CLPM

Model	χ^2^	*df*	*CFI*	TLI	RMSEA
General psychopathology, age 8	2353.365***	1,173	.93	.92	.03
General psychopathology, age 9	2144.701***	1,173	.93	.93	.03
General psychopathology, age 10	2290.673***	1,173	.93	.92	.03
General psychopathology, age 11	2413.544***	1,173	.92	.91	.03
Model 1	26.16*	15	1.00	.99	.03
Model 2	16.79	15	1.00	1.00	.01
Model 3	103.97*	75	1.00	.99	.02
Model 4	96.37*	75	1.00	.99	.02

*p < .05; ***p < .001.

### Interpersonal outcomes and the p‐factor (Models 1 and 2)

**Figure 1:**
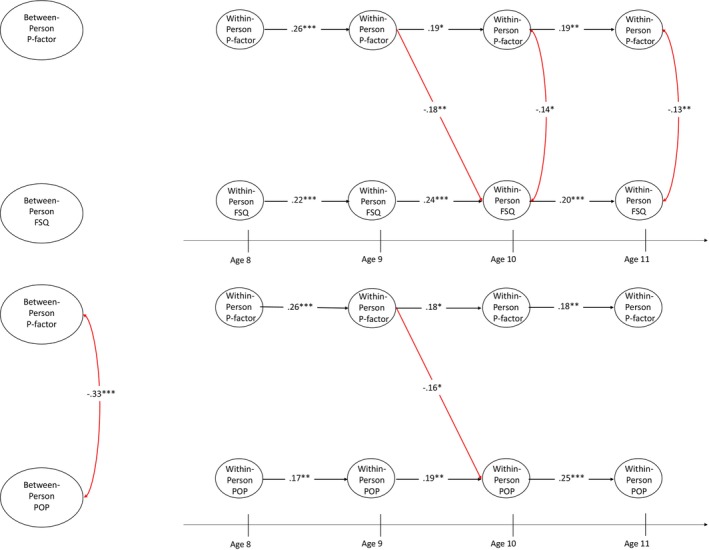
RI‐CLPM of p‐factor and friendship quality (FSQ; top panel) and popularity (POP; bottom panel) from ages 8 to 11. ATT, specific attention factor; EXT, specific externalising factor; FSQ, friendship quality; INT, specific internalising factor; POP, popularity; *p < .05; **p < .01; ***p < .001; black paths represent significant autoregressive effects, and red paths indicate significant negative effects

At the between‐person level in Model 1 (Figure 1, top panel, left), friendship quality and p‐factor were not associated. At the within‐person level (Figure 1, top panel, right), higher‐than‐expected p‐factor scores at age 9 predicted lower‐than‐expected friendship quality at age 10 (β = −.18, *p* < .01, 95% CI [−0.31, −0.06]). Additionally, deviations in friendship quality and p‐factor scores were negatively concurrently correlated with each other at ages 10 (β = −.14, *p* < .05, 95% CI [−0.26, −0.02]) and 11 (β = −.13, *p* < .01, 95% CI [−0.21, −0.05]). No other significant longitudinal pathways were identified.

At the between‐person level in Model 2 (Figure 1, bottom panel, left), perceived popularity and p‐factor scores were negatively associated (β = −.33, *p* < .001, 95% CI [−0.43, −0.24]). At the within‐person level, higher‐than‐expected p‐factor scores at age 9 predicted lower‐than‐expected popularity at age 10 (β = −.16, *p* < .05, 95% CI [−0.28, −0.04]). No other significant longitudinal pathways were identified.

Please see Appendices S4–S8 for all standardised between‐ and within‐person level effects for Models 1 and 2.

### Interpersonal outcomes and p‐factor, internalising, externalising and attention problems (Models 3 and 4)

**Figure 2:**
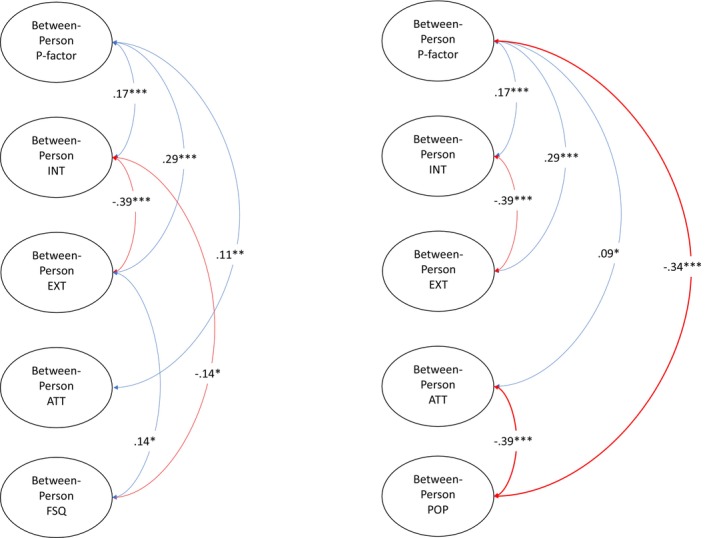
Between‐person level association between general psychopathology and both interpersonal outcomes. ATT, specific attention factor; EXT, specific externalising factor; FSQ, friendship quality; INT, specific internalising factor; POP, popularity. *p < .05, **p < .01, ***p < .001. Blue paths represent significant positive correlation, and red paths indicate significant negative correlation

**Figure 3:**
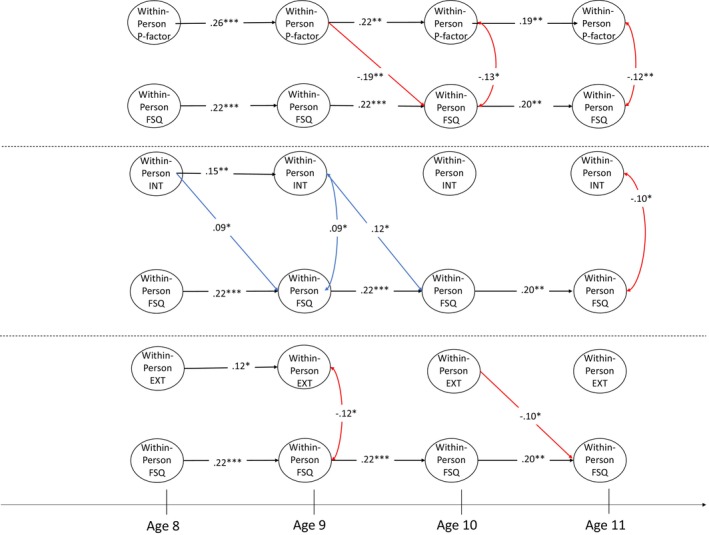
Within‐person level association between each factor of general psychopathology and friendship quality. ATT, specific attention factor; EXT, specific externalising factor; FSQ, friendship quality; INT, specific internalising factor; POP, popularity. *p < .05, **p < .01, ***p < .001. Black paths represent significant autoregressive effects, red paths represent significant negative effects, and blue paths represent significant positive effects

**Figure 4:**
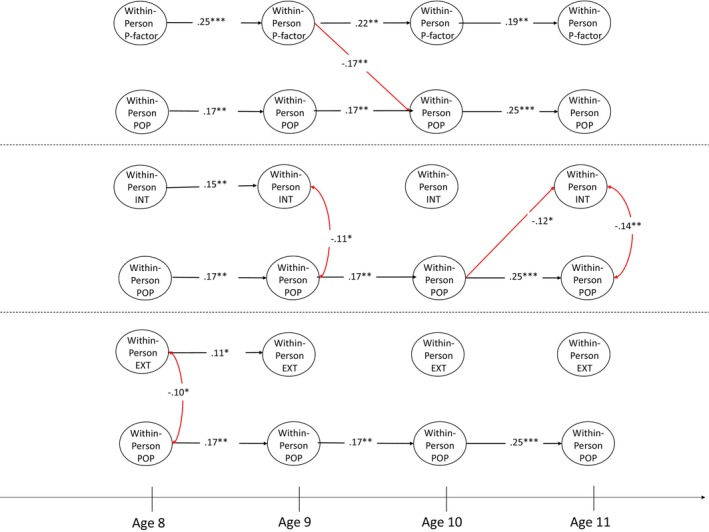
Within‐person level association between each factor of general psychopathology and friendship quality. ATT, specific attention factor; EXT, specific externalising factor; FSQ, friendship quality; INT, specific internalising factor; POP, popularity. *p < .05, **p < .01, ***p < .001. Black paths represent significant autoregressive effects, and red paths indicate significant negative effects

We further included the specific internalising, externalising and attention factor scores in Models 3 (friendship quality) and 4 (popularity). For ease of interpretation, the two models are presented in three figures. Figure 2 shows the between‐person level results, whereas Figures 3 and 4 show the within‐person level results. The within‐person level associations among specific factors of psychopathology are omitted in Figures 3 and 4 because these were not the subject of our hypotheses but can be found in Supporting Information for reference (Appendices S2 and S3).

At the between‐person level in Model 3 (Figure 2, left panel), friendship quality was associated negatively with the specific internalising factor (β = −.14, *p* < .05, 95% CI [−0.26, −0.02]) and positively with the externalising factor (β = .14, *p* < .05, 95% CI [0.02, 0.26]). At the within‐person level (Figure 3), higher‐than‐expected p‐factor scores at age 9 predicted lower‐than‐expected friendship quality at age 10 (top panel; β = −.19, *p* < .01, 95% CI [−0.31, −0.07]). Additionally, positive cross‐lagged effects from specific internalising factor scores (mid panel) at ages 8 (β = .09, *p* < .05, 95% CI [0.002, 0.18]) and 9 (β = .12, *p* < .05, 95% CI [0.03, 0.21]) to friendship quality 1 year later were found, while higher‐than‐expected specific externalising factor scores at age 10 predicted lower‐than‐expected friendship quality at age 11 (bottom panel; β = −.10, *p* < .05, 95% CI [−0.19, −0.01]). No other significant longitudinal pathways between psychopathology and friendship quality were identified.

At the between‐person level in Model 4 (Figure 2, right panel), perceived popularity was negatively associated with p‐factor (β = −.34, *p* < .001, 95% CI [−0.43, −0.31]) and with specific attention factor scores (β = −.39, *p* < .001, 95% CI [−0.50, −0.28]). At the within‐person level (Figure 4), higher‐than‐expected p‐factor scores at age 9 predicted lower‐than‐expected popularity at age 10 (top panel; β = −.17, *p* < .01, 95% CI [−0.29, −0.05]) and higher‐than‐expected popularity at age 10 predicted lower‐than‐expected specific internalising factor scores at age 11 (mid panel; β = −.12, *p* < .05, 95% CI [−0.23, −0.003]). No other significant longitudinal pathways between psychopathology and popularity were identified.

Please see Appendices S4 and S9–S12 for all standardised between‐ and within‐person level effects for Models 3 and 4.

## Discussion

Interpersonal outcomes and mental health problems are closely associated in children and preadolescents. However, most studies did not directly test their reciprocal associations and have not examined whether these associations represent the impact of and on general versus more specific aspects of psychopathology. We addressed these shortcomings by estimating random intercept cross‐lagged panel models (RI‐CLPM) to investigate the longitudinal relationships between important aspects of peer relationships (friendship quality and perceived popularity) and psychopathology (p‐factor and specific internalising, externalising and attention problems) between ages 8 and 11. Our hypotheses that negative between‐ and within‐person effects would emerge were partially supported. First, at the between‐person level, self‐reported friendship quality was associated negatively with mother‐reported internalising but *positively* with externalising problems scores; moreover, teacher‐reported child popularity was quite strongly and negatively associated with mother‐reported p‐factor and specific attention factor scores. Second, at the within‐person level, higher‐than‐expected p‐factor scores at age 9 predicted lower‐than‐expected interpersonal outcomes at age 10; moreover, higher‐than‐expected specific externalising factor scores at age 10 also predicted lower‐than‐expected friendship quality at age 11. However, contrary to our hypotheses, higher‐than‐expected specific internalising factor scores at ages 8 and 9 predicted *higher*‐than‐expected friendship quality 1 year later. Finally, as predicted, higher‐than‐expected popularity at age 10 predicted lower‐than‐expected specific internalising factor scores at age 11.

Hypothesis 1 Negative bidirectional associations between interpersonal outcomes and mental health problems at the between‐person level.

Between‐person level results suggest that preadolescents who had higher internalising and externalising problem scores also reported, on average, lower and *higher* friendship quality, respectively. Children with internalising problems may report lower friendship quality because they tend to experience more unstable friendships that are more prone to break down and they are also more pessimistic about their friendship quality (Brendgen, Vitaro, Turgeon, & Poulin, 2002; Chan & Poulin, 2009; Van Zalk, Kerr, Branje, Stattin, & Meeus, 2010). Contrary to our hypothesis, however, specific externalising factor scores were positively associated with friendship quality. Children with externalising behaviours (e.g. aggression) have been found to have excessively positive perceptions about their friendship quality, though such positive views are not necessarily reciprocated by their friends or shared by their teachers (Brendgen et al., 2002; Hoza, Pelham Jr., Dobbs, Owens, & Pillow, 2002). Also contrary to our predictions, friendship quality appeared to be less associated with p‐factor scores than anticipated at the between‐person level. One possibility is that these associations may become more stable later in development. This is consistent with Models 1 and 3, where deviations in these variables were negatively associated only at later timepoints (ages 10 and 11).

Consistent with the literature, popularity was negatively associated with p‐factor and specific attention factor scores. High p‐factor scores could relate to traits such as neuroticism and impulsivity (Southward, Cheavens, & Coccaro, 2023) which are negatively associated with popularity in children and adolescents (Spinrad et al., 2006; Van der Linden, Scholte, Cillessen, te Nijenhuis, & Segers, 2010). This is consistent with the incidental model (Parker & Asher, 1987), which posits that behaviours underlying psychopathology could emerge early and lead to later adjustment difficulties (McDonald & Gibson, 2017). Moreover, popularity was negatively associated with specific attention factor scores. This is consistent with the observation that children with ADHD tend to be less prosocial (Arango‐Tobón et al., 2023) and have less regard for fairness and rules (Normand et al., 2011), which are associated with peer problems and *un*popularity. Thus, traits and behaviours associated with mental health problems could help explain these between‐person level findings.

Hypothesis 2 Negative cross‐lagged effects from mental health problems to interpersonal outcomes.

At the within‐person level, we found partial support for our hypothesis that psychopathology (p‐factor and specific factors) would negatively predict interpersonal outcomes. Across all four models, higher‐than‐expected mother‐reported p‐factor scores at age 9 predicted lower‐than‐expected self‐rated friendship quality and teacher‐rated popularity at age 10, although not at other timepoints. The fact that these scores were reported by different informants suggests that these associations are nontrivial. Here, p‐factor at age 14 has been found to predict lower friendship quality at age 15 (Deutz et al., 2020), but our results show that this effect could emerge even earlier in preadolescence.

Also consistent with our hypothesis, higher‐than‐expected specific externalising factor scores at age 10 predicted lower‐than‐expected friendship quality at age 11. Here, children with externalising problems may have poor social skills (Bornstein et al., 2010) or be aggressive problem solvers (Salvas et al., 2011), which could hinder the acquisition and maintenance of best friendships as they transition into middle school. Taking into account the positive associations between specific externalising factor scores and friendship quality at the between‐person level, our results suggest that preadolescents with externalising problems may overestimate their best friendship quality in general across this period, but still experience friendship problems from time to time, especially as they transition into middle school between ages 10 and 11.

One unexpected finding here was that higher‐than‐expected specific internalising factor scores at ages 8 and 9 were associated with *higher‐*than‐expected deviations in friendship quality 1 year later. This contradicted our between‐person level results (specific internalising factor scores were, on average, associated with lower friendship quality across these ages) and those from previous studies (e.g. Bernasco et al., 2021). One possibility is that, at least in earlier timepoints, children experiencing internalising problems such as depression could engage in co‐rumination that has been found to associate with positive friendship quality (Felton, Cole, Havewala, Kurdziel, & Brown, 2019); alternatively, Dietvorst et al. (2018) have also used Simpson's Paradox (Kievit, Frankenhuis, Waldorp, & Borsboom, 2013) to explain discrepancy across between‐ and within‐person findings. For instance, stable levels of internalising problems can correlate negatively with friendship quality at the between‐person level across this period; however, immediately following periods of emotional problems, children could perceive their friendship quality as more positive after receiving support.

In sum, while behaviours associated with externalising problems are associated with worse interpersonal outcomes, those associated with internalising problems (e.g. co‐rumination) could be associated with higher self‐reported friendship quality.

Hypothesis 3 Negative cross‐lagged effects from friendship quality and popularity to mental health problems.

Consistent with previous findings, negative cross‐lagged effects between popularity at age 10 and specific internalising factor scores at age 11 were found (Litwack et al., 2012). Popular children may experience fewer internalising symptoms because they have more high‐quality friendships and lower levels of loneliness (Nangle, Erdley, Newman, Mason, & Carpenter, 2003), or have higher self‐esteem and feel more socially successful (Prinstein & La Greca, 2002; Sandstrom & Cillessen, 2006). This is expected as being popular is an important goal for adolescents, valued even above friendship, achievement and romantic relationships (LaFontana & Cillessen, 2002).

### Implications

The present findings have practical implications for future research and intervention programs for preadolescents. First, seemingly conflicting findings emerged between specific internalising factor scores and friendship quality at the between‐ and within‐person levels. This highlights the importance of modelling within‐person effects in future studies, as the strength and direction of effects could be different at these levels (Dietvorst et al., 2018). Examining within‐person effects is especially important if the processes to be examined are within‐person in nature (Masselink et al., 2018). Second, effects from mental health problems to interpersonal outcomes were more readily observed than the reverse. This suggests that early interventions targeting mental health problems have more potential to improve children's interpersonal outcomes.

### Strengths and limitations

The present study has three main strengths. First, measurements from mother, study child and teacher were included, which means the observed effects were not inflated by shared method variance. Second, using RI‐CLPM, we separated stable, between‐person effects from dynamic, within‐person effects, providing a more nuanced framing of the interplay between interpersonal outcomes and psychopathology. In the case of specific internalising and externalising factors, we found that their associations with friendship quality were different at these levels. Finally, the present study included a bifactor model of psychopathology to account for prior research findings and general vulnerability to mental health problems.

The current findings should also be interpreted in the context of five limitations. First, as mentioned previously, our study may be underpowered (Masselink et al., 2018), so future studies could test these reciprocal associations and explore gender differences, other moderators and non‐linear relationships with larger samples as the current study is not sufficiently powered to do so. Second, while peer report is the most common sociometric method (Cillessen, 2009), teacher‐reported popularity was used. However, Van den Berg, Lansu, and Cillessen (2015) have found considerable overlap between peer‐ and teacher‐reported popularity and suggested that teacher reports are also viable sociometric methods. Third, while the initial sample was diverse, participating families had above‐average income and education and had a lower probability to be of ethnic minorities (Watamura et al., 2011). Additionally, selective attrition could occur to more at‐risk families, potentially causing an under‐representation of children with poorer interpersonal outcomes and mental health. However, while the prevalence rates of these problems could be reduced in this scenario, the associations may nonetheless remain intact (Wolke et al., 2009). This suggests that the observed estimates could be more conservative than the likely true effects. Fourth, the current data contains only measurements of perceived but not *sociometric* popularity (i.e. being liked). We were therefore unable to test whether it is associated with mental health problems differently. Finally, data on interpersonal outcomes were collected over 20 years ago, before social media and electronic communication became commonplace. However, these recent developments could make friendship outcomes more salient and associate more strongly with mental health by allowing children consistent contact with friends even outside of school (Schwartz‐Mette et al., 2020). This means our findings could be more conservative than those based on more recent data.

## Conclusion

Our results suggest that the relationship between interpersonal outcomes and psychopathology is primarily driven by the latter, but the influences of friendship quality and popularity could become more salient later in development. These associations can also be different at the between‐ and within‐person levels.

**Key points**

- Interpersonal outcomes such as friendship quality and perceived popularity are closely associated with mental health problems in preadolescence.
- On average, preadolescent children with higher perceived popularity, but not friendship quality, had lower p‐factor scores (i.e. negative between‐person association).
- The majority of cross‐lagged effects observed at the within‐person level were negative and were from mental health problems to interpersonal outcomes.
- The associations between interpersonal outcomes and psychopathology during preadolescence may be primarily driven by the latter.

## Supporting information


Appendix S1.
**Table S1.** Supplementary description for bifactor models
**Table S2.** Standardised between‐person correlation for all models.
**Table S3.** Standardised within‐person autoregressive and cross‐lagged effects for Model 1.
**Table S4.** Standardised within‐person correlation for Model 1.
**Table S5.** Standardised within‐person autoregressive and cross‐lagged effects for Model 2.
**Table S6.** Standardised within‐person correlation for Model 2.
**Table S7.** Standardised within‐person autoregressive and cross‐lagged effects for Model 3.
**Table S8.** Standardised within‐person correlation for Model 3.
**Table S9.** Standardised within‐person autoregressive and cross‐lagged effects for Model 4.
**Table S10.** Standardised within‐person correlation for Model 4.
**Figure S1.** RI‐CLPM of general psychopathology (p‐factor and specific factors) and friendship quality (FSQ) from ages 8 to 11.
**Figure S2.** RI‐CLPM of general psychopathology (p‐factor and specific factors) and popularity (POP) from ages 8 to 11.

## Data Availability

Data used in this study came from the Study of Early Child Care and Youth Development (SECCYD). Researchers can request access to the SECCYD data: https://www.nichd.nih.gov/research/supported/seccyd/overview#instruments.
