# Supplementary material for: Examining longitudinal associations between interpersonal outcomes and general psychopathology factors across preadolescence using random intercept cross‐lagged panel model
Source: J Child Psychol Psychiatry. 2024 Dec 28;66(7):932–45. doi: 10.1111/jcpp.14105 (PMC12198929; doi:10.1111/jcpp.14105)
Supplement: Supplementary file 1 — Appendix S1. Table S1. Supplementary description for bifactor models Table S2. Standardised between‐person correlation for all models. Table S3. Standardised within‐person autoregressive and cross‐lagged effects for Model 1. Table S4. Standardised within‐person correlation for Model 1. Table S5. Standardised within‐person autoregressive and cross‐lagged effects for Model 2. Table S6. Standardised within‐person correlation for Model 2. Table S7. Standardised within‐person autoregressive and cross‐lagged effects for Model 3. Table S8. Standardised within‐person correlation for Model 3. Table S9. Standardised within‐person autoregressive and cross‐lagged effects for Model 4. Table S10. Standardised within‐person correlation for Model 4. Figure S1. RI‐CLPM of general psychopathology (p‐factor and specific factors) and friendship quality (FSQ) from ages 8 to 11. Figure S2. RI‐CLPM of general psychopathology (p‐factor and specific factors) and popularity (POP) from ages 8 to 11. [file JCPP-66-932-s001.docx]

**Supplementary Table ST1.** Supplementary description for bifactor models

Goodness-of-fit of the bifactor models was further assessed using model-based reliability (ω), explained common variance (ECV), and *H*. First, the general p-factor and the specific factors at all four timepoints demonstrated good-to-excellent model-based reliability (ω= .88- .97). Second, p-factor consistently explained the majority of variance at all four timepoints (ECV= .63-.67), compared to specific internalising (ECV= .42-.51), externalizing (ECV= .22-.25), and attention (ECV= .41-.42) factors. To note, as the ECV values for the p-factor were consistently below .70, the variance explained by the specific factors can be considered nontrivial (Rodriguez et al., 2016). Finally, *H* values were consistently high for the p-factor (*H*= .96-.97) and specific internalising factor (*H*= .83-.87). On the other hand, *H* values ranged from .75 to .78 for specific externalising factor and from .69 to .73 for specific attention factor, suggesting that these factors were less well-defined (Hancock & Mueller, 2001).

**Table 1** Bifactor-specific indices across time

| Model | ECV | Omega | H |
| --- | --- | --- | --- |
| GPF, age 8 | .628 | .969 | .963 |
| INT, age 8 | .507 | .931 | .867 |
| EXT, age 8 | .236 | .953 | .780 |
| ATT, age 8 | .412 | .898 | .721 |
| GPF, age 9 | .663 | .969 | .963 |
| INT, age 9 | .395 | .930 | .827 |
| EXT, age 9 | .249 | .949 | .775 |
| ATT, age 9 | .420 | .899 | .728 |
| GPF, age 10 | .672 | .970 | .965 |
| INT, age 10 | .424 | .931 | .851 |
| EXT, age 10 | .222 | .953 | .768 |
| ATT, age 10 | .375 | .892 | .688 |
| GPF, age 11 | .662 | .969 | .964 |
| INT, age 11 | .440 | .934 | .846 |
| EXT, age 11 | .217 | .951 | .752 |
| ATT, age 11 | .406 | .884 | .701 |

*Note.* ECV= Explained common variance

**Supplementary Figure SF1** RI-CLPM of general psychopathology (p-factor and specific factors) and friendship quality (FSQ) from ages 8 to 11


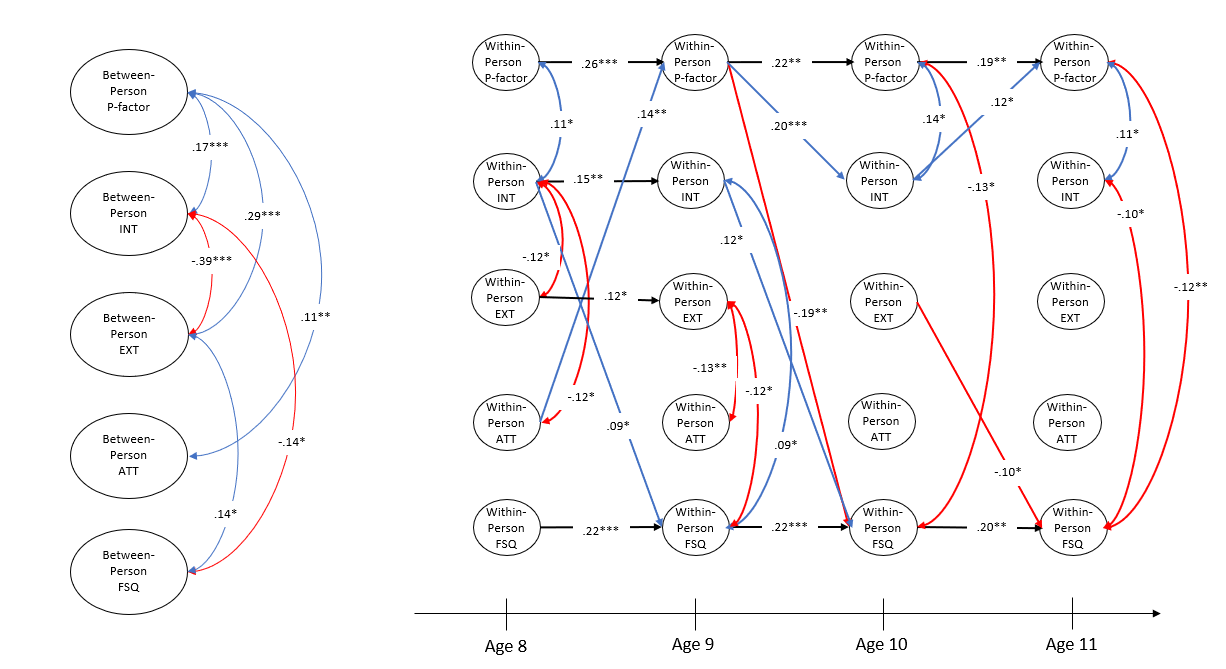


*Note.* INT= specific internalising factor; EXT= specific externalising factor; ATT= specific attention factor; FSQ= friendship quality; POP= popularity; *p< .05; **p< .01; ***p< .001; black paths = significant autoregressive effects; red paths = significant negative effects; blue paths = significant positive effects

**Supplementary Figure SF2** RI-CLPM of general psychopathology (p-factor and specific factors) and popularity (POP) from ages 8 to 11


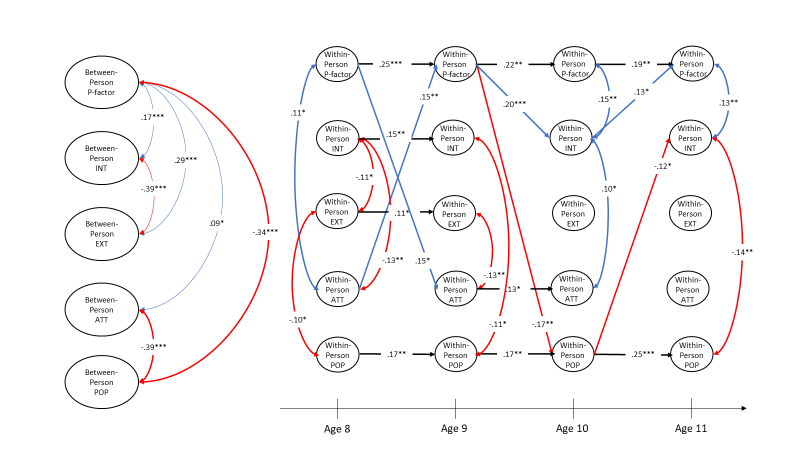


*Note.* INT= specific internalising factor; EXT= specific externalising factor; ATT= specific attention factor; FSQ= friendship quality; POP= popularity; *p< .05; **p< .01; ***p< .001; black paths = significant autoregressive effects; red paths = significant negative effects; blue paths = significant positive effects

**Supplementary Table ST2.** Standardised between-person correlation for all models

|  | Model 1  [95% CI] | Model 2  [95% CI] | Model 3  [95% CI] | Model 4  [95% CI] |
| --- | --- | --- | --- | --- |
| **Effects with P-factor** |  |  |  |  |
| **INT** |  |  | **.17***, [.10, .25]** | **.17***, [.09, .25]** |
| **EXT** |  |  | **.29***, [.22, .36]** | **.29***, [.22, .36]** |
| **ATT** |  |  | **.11**, [.03, .18]** | **.09*, [.02, .17]** |
| **FSQ** | -.09 [-.20, .02] |  | -.09, [-.20, .20] |  |
| **POP** |  | **-.33*** [-.43, -.24]** |  | **-.34***, [-.43, -.25]** |
| **Effects with INT** |  |  |  |  |
| **EXT** |  |  | **-.39***, [-.47, -.31]** | **-.39***, [-.48, -.31]** |
| **ATT** |  |  | -.05, [.14, .05] | -.05, [-.14, .04] |
| **FSQ** |  |  | **-.14*, [-.26, -.02]** |  |
| **POP** |  |  |  | -.03, [-.14, .07] |
| **Effects with EXT** |  |  |  |  |
| **ATT** |  |  | .04, [-.06, .14] | .04, [-.06, .14] |
| **FSQ** |  |  | **.14*, [.02, .26]** |  |
| **POP** |  |  |  | -.02, [-.13, .09] |
| **Effects with ATT** |  |  |  |  |
| **FSQ** |  |  | -.06, [.17, .06] |  |
| **POP** |  |  |  | **-.39***, [-.50, -.28]** |

*Note.* **p*< .05; ***p*< .01; ****p*< .001

**Supplementary Table ST3.** Standardised within-person autoregressive and cross-lagged effects for Model 1

|  | Age 8 to 9, [95% CI] | Age 9 to 10, [95% CI] | Age 10 to 11, [95% CI] |
| --- | --- | --- | --- |
| **Effects on p-factor** |  |  |  |
| **p-factor** | **.26***, [.14, .38]** | .19*, [.04, .33] | **.19**, [.06, .32]** |
| **FSQ** | .03, [-.06, .12] | .06, [-.06, .18] | -.08, [-.19, .03] |
| **Effects on FSQ** |  |  |  |
| **p-factor** | -.01, [-.12, .11] | **-.18**, [-.31, -.06]** | -.04, [-.13, .06] |
| **FSQ** | **.22***, [.12, .32]** | **.24***, [.13, .34]** | **.20***, [.09, .31]** |

*Note.* **p*< .05; ***p*< .01; ****p*< .001

**Supplementary Table ST4**. Standardised within-person correlation for Model 1

|  | Age 8,  [95% CI] | Age 9,  [95% CI] | Age 10,  [95% CI] | Age 11,  [95% CI] |
| --- | --- | --- | --- | --- |
| **Effects with p-factor** |  |  |  |  |
| **FSQ** | -.02, [-.07, .12] | -.03, [-.14, .08] | **-.14*, [-.26, -.02]** | **-.13**, [-.21, -.05]** |

*Note.* **p*< .05; ***p*< .01; ****p*< .001

**Supplementary Table ST5.** Standardised within-person autoregressive and cross-lagged effects for Model 2

|  | Age 8 to 9  [95% CI] | Age 9 to 10  [95% CI] | Age 10 to 11  [95% CI] |
| --- | --- | --- | --- |
| **Effects on p-factor** |  |  |  |
| **p-factor** | **.26***, [.14, .38]** | **.18*, [.04, .33]** | **.18**, [.05, .32]** |
| **POP** | -.03, [-.13, .07] | .01, [-.12, .14] | -.06, [-.17, .05] |
| **Effects on POP** |  |  |  |
| **p-factor** | .06, [-.07, .18] | **-.16*, [-.28, -.04]** | .08, [-.02, .19[ |
| **POP** | **.17**, [.05, .29]** | **.19**, [.07, .32]** | **.25***, [.15, .36]** |

*Note.* **p*< .05; ***p*< .01; ****p*< .001

**Supplementary Table ST6.** Standardised within-person correlation for Model 2

|  | Age 8  [95% CI] | Age 9  [95% CI] | Age 10  [95% CI] | Age 11  [95% CI] |
| --- | --- | --- | --- | --- |
| **Effects with p-factor** |  |  |  |  |
| **POP** | -.10, [-.21, .01] | -.06, [-16, .05] | -.08, [-.20, .04] | .04. [-.06, .15] |

*Note.* **p*< .05; ***p*< .01; ****p*< .001

**Supplementary Table ST7.** Standardised within-person autoregressive and cross-lagged effects for Model 3

|  | Age 8 to 9  [95% CI] | Age 9 to 10  [95% CI] | Age 10 to 11  [95% CI] |
| --- | --- | --- | --- |
| **Effects on p-factor** |  |  |  |
| **p-factor** | **.26***, [.13, .38]** | **.22**, [.08, .34]** | **.19**, [.07, .32]** |
| **INT** | .09, [-.02, .19] | -.01, [-.13, .11] | **.12*, [.02, .23]** |
| **EXT** | .03, [-.07, .12] | .04, [-.07, .15] | .07, [-.02, .17] |
| **ATT** | **.14**, [.03, .24]** | .02, [-.09, .13] | -.02, [-.13, .09] |
| **FSQ** | .02, [-.07, .11] | .07, [-.05, .19] | -.08, [-.19, .02] |
| **Effects on FSQ** |  |  |  |
| **p-factor** | -.01, [-.12, .10] | **-.19**, [-.31, -.07]** | -.04, [-.14, .06] |
| **INT** | **.09*, [.002, .18]** | **.12*, [.03, .21]** | .04, [-.05, .14] |
| **EXT** | -.06, [-.15, .02] | -.03, [-.12, .07] | **-.10*, [-.19, -.01]** |
| **ATT** | -.004, [-.09, .08] | .01, [-.08, .09] | .01, [-.08, .11] |
| **FSQ** | **.22***, [.12, .31]** | **.22***, [.12, .33]** | **.20***, [.09, .31]** |
| **Effects on INT** |  |  |  |
| **p-factor** | -.03, [-.15, .09] | **.20***, [.09, .31]** | .07, [-.05, .19] |
| **INT** | **.15**, [.04, .25]** | .06, [-.06, .17] | .06, [-.05, .18] |
| **EXT** | .001<, [-.09, .09] | -.02, [-.13, .08] | -.01, [-.10, .09] |
| **ATT** | .001<, [-.11, .11] | -.06, [-.15, .04] | .03, [-.08, .14] |
| **FSQ** | .08, [-.01, .16] | .08, [-.02, .18] | .003, [-.11, .11] |
| **Effects on EXT** |  |  |  |
| **p-factor** | .11, [-.01, .22] | -.05, [-.16, .06] | -.07, [-.19, .06] |
| **INT** | -.05, [-.15, .04] | .10, [-.01, .21] | .10, [-.02, .21] |
| **EXT** | **.12*, [.01, .22]** | .04, [-.07, .16] | -.02, [-.14, .10] |
| **ATT** | .01, [-.10, .11] | -.08, [-.18, .02] | .06, [-.18, .05] |
| **FSQ** | -.01, [-.09, .08] | -.08, [-.17, .02] | .07, [-.18, .05] |
| **Effects on ATT** |  |  |  |
| **p-factor** | .10, [-.03, .22] | -.01, [-.14, .12] | .07, [-.04, .17] |
| **INT** | -.05, [-.15, .05] | .05, [-.06, .17] | -.06, [-.16, .03] |
| **EXT** | -.06, [-.15, .04] | -.07, [-.18, .04] | -.03, [-.13, .06] |
| **ATT** | .07, [-.05, .19] | .11, [-.01, .23] | .10, [-.01, .20] |
| **FSQ** | -.04, [-.13, .05] | -.02, [-.13, .08] | .01, [-.09, .10] |

*Note.* **p*< .05; ***p*< .01; ****p*< .001

**Supplementary Table ST8.** Standardised within-person correlation for Model 3

|  | Age 8  [95% CI] | Age 9  [95% CI] | Age 10  [95% CI] | Age 11  [95% CI] |
| --- | --- | --- | --- | --- |
| **Effects with p-factor** |  |  |  |  |
| **INT** | **.11*, [.01, .22]** | .09, [-.02, .20] | **.14**, [.04, .25]** | **.11*, [.02, .20]** |
| **EXT** | .1, [-.002, .20] | .04, [-.06, .13] | -.05, [-.15, .06] | -.05, [-.15, .05] |
| **ATT** | -.01, [-.12, .09] | -.04, [-.15, .07] | -.10, [-.22, .06] | -.05, [-.15, .04] |
| **FSQ** | .02, [-.08, .11] | -.03, [-.13, .08] | **-.13*, [-.25, -.01]** | **-.12**, [-.20, -.04]** |
| **Effects with FSQ** |  |  |  |  |
| **INT** | .01, [-.08, .10] | **.09*, [.01, .18]** | .08, [-.01, .17] | **-.10*, [-.19, .01]** |
| **EXT** | .02, [-.06, .11] | **-.12*, [-.20, -.03]** | -.05, [-.15, .06] | .02, [-.07, .11] |
| **ATT** | .03, [-.07, .13] | -.02, [-.12, .07] | -.08, [-.18, .02] | .02, [-.07, .11] |
| **Effects with INT** |  |  |  |  |
| **EXT** | -.12*, [-.21, -.03] | .02, [-.07, .11] | -.01, [-.12, .10] | -.05, [-.14, .05] |
| **ATT** | -.12*, [-.22, -.02] | .02, [-.09, .13] | .09, [-.01, .19] | -.03, [-.12, .07] |
| **Effects with EXT** |  |  |  |  |
| **ATT** | .05, [-.04, .15] | **-.13**, [-.23, -.04]** | -.09, [-.19, .02] | .03, [-.07, .12] |

*Note.* **p*< .05; ***p*< .01; ****p*< .001

**Supplementary Table ST9.** Standardised within-person autoregressive and cross-lagged effects for Model 4

|  | Age 8 to 9  [95% CI] | Age 9 to 10  [95% CI] | Age 10 to 11  [95% CI] |
| --- | --- | --- | --- |
| **Effects on p-factor** |  |  |  |
| **p-factor** | **.25***, [.13, .37]** | **.22**, [.09, .35]** | **.19**, [.06 , .32]** |
| **INT** | .10, [-.01, .20] | -.01, [-.12, .11] | **.13*, [.02, .23]** |
| **EXT** | .03, [-.06, .13] | .03, [-.07, .13] | .07, [-.03, .16] |
| **ATT** | **.15**, [.05, .25]** | .04, [-.07, .15] | -.02, [-.13, .09] |
| **POP** | -.03, [-.13, .07] | .02, [-.11, .14] | -.06, [-.16, .05 |
| **Effects on INT** |  |  |  |
| **p-factor** | -.04, [-.16, .08] | **.20***, [.10, .31]** | .07, [-.05, .19] |
| **INT** | **.15**, [.04, .26]** | .07, [-.05, .18] | .06, [-.06, .17] |
| **EXT** | .01, [-.09, .10] | -.03, [-.13, .08] | -.001, [-.10, .10] |
| **ATT** | .003, [-.11, .12] | -.07, [-.16, .02] | .05, [-.06, .16] |
| **POP** | .02, [-.08, .11] | -.04, [-.15, .07] | **-.12*, [-.23, -.003]** |
| **Effects on EXT** |  |  |  |
| **p-factor** | .09, [-.03, .20] | -.05, [-.15, .05] | -.07, [-.20, .06] |
| **INT** | -.04, [-.13, .06] | .08, [-.03, .19] | .10, [-.01, .22] |
| **EXT** | **.11*, [.002, .22]** | .05, [-.07, .16] | -.03, [-.15, .10] |
| **ATT** | -.002, [-.11, .10] | -.07, [-.17, .03] | -.07, [-.19, .04] |
| **POP** | -.09, [-.19, .10] | -.07, [-.17, .03] | -.05, [-.16, .07] |
| **Effects on ATT** |  |  |  |
| **p-factor** | **.15*, [.02, .27]** | -.001, [-.13, .13] | .06, [-.05, .17] |
| **INT** | -.06, [-.16, .04] | .05, [-.06, .17] | -.06, [-.15, .04] |
| **EXT** | -.05, [-.14, .05] | -.07, [-.18, .04] | -.04, [-.14, .05] |
| **ATT** | .07, [-.05, .19] | **.13*, [.04, .25]** | .10, [-.01, .21] |
| **POP** | .03, [-.08, .13] | .05, [-.07, .17] | .01, [-.08, .11] |
| **Effects on POP** |  |  |  |
| **p-factor** | .08, [-.08, .21] | **-.17**, [-.29, -.05]** | .10, [-.02, .22] |
| **INT** | -.06, [-.17, .05] | -.08, [-.19, .04] | -.02, [-.12, .08] |
| **EXT** | -.04, [-.15, .06] | -.05, [-.15, .05] | .09, [-.002, -.19] |
| **ATT** | .04, [-.08, .17] | .02, [-.09, .13] | .07, [-.04,.17] |
| **POP** | **.17**, [.05, .28]** | **.17**, [.05, .30]** | **.25***, [.14, .36]** |

*Note.* **p*< .05; ***p*< .01; ****p*< .001

**Supplementary Table ST10.** Standardised within-person correlation for Model 4

|  | Age 8  [95% CI] | Age 9  [95% CI] | Age 10  [95% CI] | Age 11  [95% CI] |
| --- | --- | --- | --- | --- |
| **Effects with p-factor** |  |  |  |  |
| **INT** | -.09, [-.004, .22] | .10, [-.01, .21] | **.15**, [.04, .25]** | **.13**, [.04, .22]** |
| **EXT** | .11, [-.01, .21] | .03, [-.07, .12] | -.05, [-.16, .05] | -.06, [-.16, .04] |
| **ATT** | **.11*, [-.09, .12]** | -.01, [-.12, .11] | -.11, [-.22, .01] | -.06, [-.16, .03] |
| **POP** | .02, [-.20, .20] | -.06, [-.17, .05] | -.08, [-.20, .03] | .04, [-.07, .03] |
| **Effects with POP** |  |  |  |  |
| **INT** | .05, [-.05, .14] | **-.11*, [-.22, -.01]** | -.03, [-.13, .08] | **-.14**, [-.24, -.05]** |
| **EXT** | **-.10*, [-.19, -.004]** | -.03, [-.13, .07] | .02, [-.09, .12] | .04, [-.06, .13] |
| **ATT** | -.08, [-.19, .03] | -.07, [-.18, .05] | .07, [-.31, .17] | .09, [-.01, .19] |
| **Effects with INT** |  |  |  |  |
| **EXT** | **-.11*, [-.20, .02]** | .03, [-.07, .12] | -.01, [-.12, .10] | -.05, [-.14, .05] |
| **ATT** | **-.13*, [-.23, -.02]** | .01, [-.09, .12] | **.10*, [.001, .20]** | -.02, [-.12, .08] |
| **Effects with EXT** |  |  |  |  |
| **ATT** | .07, [-.03, .16] | **-.13*, [-.22, -.03]** | -.09, [-.20, .20] | .01, [-.09, .11] |

*Note.* **p*< .05; ***p*< .01; ****p*< .001
